# Vetiver, *Vetiveria zizanioides* (L.) Nash: Biotechnology, Biorefineries, and the Production of Volatile Phytochemicals

**DOI:** 10.3390/plants14101435

**Published:** 2025-05-10

**Authors:** Ian G. C. Barcellos-Silva, Filipe K. F. dos Santos, Harsha Kharkwal, Subhash Chander, Amit C. Kharkwal, Rajendra Awasthi, Neerupma Dhiman, Bhupesh Sharma, Giriraj T. Kulkarni, Harold Larssen, Jôsy M. L. Silva, Márcio A. de Souza, William N. Setzer, Valdir F. Veiga-Junior

**Affiliations:** 1 Military Institute of Engineering, Rio de Janeiro, Praça General Tibúrcio, 80, Praia Vermelha, Urca, Rio de Janeiro 22290-270, Brazil; iangardel123@gmail.com (I.G.C.B.-S.); filipe.kayode@gmail.com (F.K.F.d.S.); leite.jose@ime.eb.br (J.M.L.S.); marcimaraujodesouza@ime.eb.br (M.A.d.S.); 2 Amity Institute of Phytochemistry and Phytomedicine, Amity University Uttar Pradesh, Noida 201313, India; 3 Amity Institute of Pharmacy, Amity Education Valley, Amity University Haryana, Panchgaon, Gurgaon 122412, India; schander@ggn.amity.edu; 4 Amity Institute of Microbial Technology, Amity University Uttar Pradesh, Noida 201313, India; ackharkwal@amity.edu; 5 Department of Pharmaceutical Sciences, School of Health Sciences and Technology, University of Petroleum and Energy Studies, Dehradun 248 007, Uttarakhand, India; awasthi02@gmail.com; 6 Amity Institute of Pharmacy, Amity University Uttar Pradesh, Sector-125, Noida 201313, India; ndhiman@amity.edu; 7 Department of Pharmaceutical Sciences, Faculty of Life Sciences, Gurugram University, Gurugram 122003, Haryana, India; drbhupeshresearch@gmail.com; 8 School of Pharmaceutical and Population Health Informatics, DIT University, Dehradun 248009, India; gtkulkarni@gmail.com; 9 International University of Applied Sciences, D-99084 Berlin, Germany; larssen.harold@gmail.com; 10 Department of Chemistry, University of Alabama in Huntsville, Huntsville, AL 35899, USA; wsetzer@chemistry.uah.edu; 11 Aromatic Plant Research Center, 230 N 1200 E, Suite 100, Lehi, UT 84043, USA

**Keywords:** phytoremediation, soil stabilization, pharmacological potential, global market, phytoconstituents

## Abstract

This current review study covers the applications of vetiver essential oil (VEO) in phytoremediation, emphasizing its remedial capabilities in the cleaning of environmental pollutants like pesticides, fertilizers, fungicides, herbicides, heavy metals, dyes, and other industrial wastes such as chemical, mining, pharmaceutical, and other radioactive wastes. The review also emphasizes the pharmacological potential of vetiver essential oil for different applications, such as antioxidant, anti-inflammatory, antifungal, antibacterial, antitubercular, antihyperglycemic, antidepressant, hepatoprotective, and nephroprotective uses. The commercial potential of vetiver essential oil in diverse sectors, including global perspectives, is also illustrated along with demand scenarios in different sectors like food, beverage, fragrance, cosmetic and aromatherapy, hygiene, and pharmaceutical sectors. The main constituents of vetiver oil, their relative proportion, and the key findings of pharmacological studies performed using VEOs or their constituents are also summarized in this review article, with special emphasis on activity against phytopathogens.

## 1. Introduction

The exploration of biodiversity is increasingly promoting the prospect of new technologies and the generation of bioproducts. Researchers look for efficiency, safety, and well-established quality standards regarding these bioproducts in order to apply them to alleviate the most diverse human and environmental health problems. The plant species *Vetiveria zizanioides* (L.) Nash (synonym *Chrysopogon zizanioides* (L.) Roberty) represents a distinct specimen widely utilized in many diverse industrial sectors. Commonly known as vetiver, this species is a member of the Poaceae family and is originally native to India and Southeast Asia but is now globally distributed in tropical and subtropical regions [1].

According to its physiological characteristics, vetiver is classified as a grass species that forms large dense clumps and is also composed of a massive spongy fibrous root system. It presents a rapid growth ratio that may reach 3.0 cm per day during the early stages of development. Its roots may descend to 4.0 m deep in the ground within a 12-month period. Its culms are robust, erect, and relatively rigid. The plant’s size varies from 1.0 to 2.5 m tall and, even though it produces inflorescences, its seeds are sterile. This characteristic provides a strategic use for this grass since it is not invasive [2].

This high adaptability of the plant allows it to endure in the most diverse environmental conditions. Its deep root system makes it resistant to short drought periods and also helps to prevent slope slippage. Vetiver presents resistance not only to temperature variations in a –15 °C to 55 °C range but is also resilient to soil pH changes from pH 3 to pH 11. Most herbicides, pesticides, pests, and diseases are also ineffective against this grass species. These characteristics allow high versatility regarding vetiver’s usage in various industrial sectors as a broad-spectrum bioremediation agent [3]. The biotechnology employed to generate vetiver plant cultivars has become increasingly refined and precise. This technological improvement takes the plant species to a robust industrial level. It also expands the possibilities for the application of its raw materials to a large number of different industrial sectors. Several examples of vetiver’s multi-purpose applications can be highlighted: environmental bioremediation in the treatment of soils contaminated by heavy metals; use in the pharmaceutical industry due to its secondary metabolites with pharmacological properties, such as antioxidants; and use in the cosmetology sector, where essential oil derived from plants of this species is the flagship in the formulation of perfumes and cosmetics with high market value [3,4].

## 2. Review Methodology

Only peer-reviewed research articles and reviews published in English-language scientific journals, book chapters, and books were included. Relevant data on chemical structures, bioactivity mechanisms, pharmacokinetics, bioavailability, and toxicity were systematically retrieved from PubMed, Google Scholar, Web of Science, Scopus, and ScienceDirect. A structured search strategy incorporating keywords was employed, including the following terms: vetiver, vetiver oil, and *Vetiveria zizanioides*. Inclusion criteria: (i) peer-reviewed original research and reviews from scientific journals, book chapters, and books, (ii) preclinical (in vitro and in vivo) and clinical studies evaluating the biological activities of *Vetiveria zizanioides* and its derivatives, (iii) studies discussing pharmacokinetics, bioavailability, and toxicity of *V. zizanioides* essential oil, extracts, and isolated components, (iv) mechanistic studies, and (v) English-language publications. Exclusion criteria: (i) non-English articles unless available in translated, peer-reviewed sources, (ii) case reports, conference abstracts, editorials, and commentaries lacking experimental data, (iii) studies unrelated to *V. zizanioides* biological activities, (iv) duplicate studies from different databases, retaining only the most recent and complete versions, and (v) studies with insufficient or inconclusive data.

## 3. Vetiver: From Countryside to Large Industries

### 3.1. Biotechnological Potential of Vetiver in Agribusiness

Environmental contamination commonly results from human activities across various industries such as food, chemical, pharmaceutical, textile, mining, nuclear, and inappropriate farming systems. Bioremediation is an emerging approach used to manage waste material originating from such industries and households using natural resources. In bioremediation, the water treatment is conducted by degrading and detoxifying contaminants through the principle of the biogeochemical cycle. Bioremediation is broadly classified as phytoremediation (plants are used to treat waste) and rhizoremediation (plants and microbes are used to treat waste) [5,6]. Bioremediation is also a sustainable and replicable approach for soil conservation [7].

The application of vetiver grass in phytoremediation is an effective approach to removing water pollutants [8]. Vetiver can grow in harsh environmental conditions and has fleshy leaves, root aroma, long roots for deep penetration, and metal adsorption capacity. Xerophytic and hydrophytic attributes allow vetiver to withstand extreme weather conditions. Thus, these characteristics make it an ideal candidate for bioremediation applications [9]. Vetiver is mainly used in bioremediation research for disaster risk management like the control of oil and mining spills, the treatment of garbage dumps, pesticide and agrochemical removal, the absorption of heavy metals, water purification, effluent treatments, and nuclear waste treatment [10,11]. Fonseca Largo et al. designed and constructed PVC pipe-based artificial floating islands with vetiver for the removal of geogenic arsenic from Ilinizas Reservoir in Ecuador. The study reported an average arsenic remediation of 97% in both water and sediment. On the other hand, an average iron remediation of 87% was recorded in sediment [12]. Suelee et al. investigated the removal efficiency of heavy metals (Mn^2+^, Cu^2+^, Pb^2+^, Fe^2+^, and Zn^2+^) in water using vetiver grass. The removal efficiency was recorded in a rank order of Fe > Pb > Cu > Mn > Zn. Higher density and long root length had been associated with enhanced removal efficacy. Roots generally have higher metal uptake capacity than shoots [8]. Hailu et al. evaluated the effect of vetiver grass on crop production and soil properties in western Ethiopia. Soil samples were collected from vetiver grass-treated hedgerows and adjacent untreated cropland. Vetiver grass-treated soil had higher levels of available phosphorus and soil organic carbon than the untreated soil. However, no significant difference was recorded in the exchangeable acidity, soil pH, total nitrogen, and available potassium among the croplands. A higher mean crop yield was recorded in vetiver-treated soil than the untreated soil. Vetiver grass reduced the average slope by 7% compared to the untreated cropland [13]. Raman and Gnansounou comprehensively reviewed the applications of vetiver in the bioremediation of industrial pollutants [5].

#### 3.1.1. Vetiver for Bioremediation in Food Industries

Despite several benefits, the introduction of pesticides, fertilizers, fungicides, and herbicides in the environment causes serious health hazards. This risk has been increased with the development of modern agricultural technologies and thus several measures need to be taken to control the environmental contamination caused by food industries [14,15]. Bioremediation approaches can be helpful to clean up such environments contaminants generated from food industries. Despite the economic importance of breweries in the agro-food sector, it is a major water-pollution-causing industry. Vetiver grass grown using a hydroponics technique can be a promising alternative for the bioremediation of brewery wastewater. Worku et al. reported removal efficiency of vetiver grass up to 58% and 73% for Chemical Oxygen Demand (COD) and Biochemical Oxygen Demand (BOD), respectively. The removal efficiencies ranged from 26 to 46% for Total Kjeldahl Nitrogen (TKN), 28 to 46% for NH_4_^+^-N, 35 to 58% for NO_3_^−^-N, and 42 to 63% for PO_4_^−3^-P [16]. Vetiver can treat soil and water contaminated by atrazine (a commonly used herbicide) [17,18,19]. Atrazine detoxification involves glutathione-*S*-transferases catalysis in conjugation to glutathione [19]. Vetiver has an arsenic tolerance of up to 225 mg/kg. Datta et al. reported 10.6% arsenic removal in soil contaminated with 45 mg/kg [20].

In a greenhouse hydroponic experiment, vetiver had effectively removed prometryn (2,4-bis (isopropylamino)-6-methylthio-s-triazine), a common pesticide used in the cultivation of vegetables, rice, wheat, and cotton. Vetiver shortened the half-life of prometryn by 11.5 days [21,22]. Vetiver was shown to effectively remove endosulfan (an organochlorine insecticide) [23] from the contaminated planted and unplanted soils (K_f_ = 6.53–9.73 mg^1–n^L^n^kg^−1^ and 6.27–7.24 mg^1–n^L^n^kg^−1^, respectively) [24]. Excess quantity of any fertilizer in the soil contaminates the environment through runoff and erosion. Boron, a toxic fertilizer, is essential for the growth of plants. It can be removed through a bioremediation technique using vetiver [25,26,27]. Vetiver survived in high boron concentrations, up to 750 mg L^−1^ [27]. Water and soil are contaminated by the secondary effluent generated during palm oil production in the palm industry. Bioremediation using vetiver is a cost-effective technique to reduce COD and BOD from the generated effluent [28]. The remediation of wastewater from coconut husk using vetiver effectively reduces chemical oxygen demand, biological oxygen demand, electrical conductivity, and total dissolved solids [29].

#### 3.1.2. Vetiver for Bioremediation in Chemical Industries

Approximately 10 million tons of toxic chemicals are released into the environment every year. Management of such chemical waste is essential to maintain good health conditions for humans and all living beings. Chemical waste treatment by vetiver is an economical approach that has predominated over chemical bioremediation. Singh et al. investigated bioremediation of phenol using vetiver. The study reported complete removal of phenol from a solution with 50–100 mg/L and 70%, 76%, and 89% removal from 1000, 500, and 200 mg/L, respectively. The production of hydrogen peroxide and peroxidase is responsible for phenol removal during vetiver plant growth in a phenolic environment. However, phenol inhibits vetiver growth [30]. Saeb et al. reported 8 mg/kg cyanide removal using vetiver in a pH-dependent manner. The capacity increased with an increased quantity of water, pH, and growth time [31]. *Achromobacter xylosoxidans* (an endophytic bacterium) strain improves vetiver biomass growth while degrading phenol [32]. TNT (2,4,6-trinitrotoluene) prepared in explosives manufacturing sites is carcinogenic and poses serious health issues. Vetiver grass can uptake TNT [33,34]. Makris et al. reported 1.03 mg/g uptake of TNT by vetiver [34]. A higher uptake of TNT (40 mg/g) has been reported for a vetiver–urea system due to the chaotropic effect of urea [35].

#### 3.1.3. Vetiver for Bioremediation in Pharmaceutical Industries

Pharmaceutical products are a major source of chemical contamination that can be generated from pharmaceutical industries and the unsafe disposal of expired or unused drugs from clinics and households [35,36,37]. The unsafe disposal of antibiotics in soil and aquatic ecosystems may lead to antibiotic resistance [38,39,40,41,42]. Vetiver can treat water contaminated with antibiotics like tetracycline [43] through amide hydrolysis and GST-mediated conjugation [42]. Vetiver has been reported to treat soil and wastewater with the non-steroidal anti-inflammatory naproxen by bioremediation, being able to remove from 81.5% to 60.9% of naproxen at 100 to 500 mgL^−1^ concentrations [44]. Recently, Panja et al. studied the potential of vetiver to remove ciprofloxacin and tetracycline from wastewater effluent. More than 90% of the examined antibiotics were removal within 30 days. It also removed > 40%, >60%, >50%, >40% of nitrate, phosphate, total organic carbon, and chemical oxygen demand from wastewater, respectively [39].

#### 3.1.4. Vetiver for Bioremediation in Textile Industries

With the use of more than 8000 different chemicals, the textile sector is placed as the second-largest polluting industry. Bleaches, detergents, dyes, fixing agents, heavy metals, and several solvents are commonly generated from textile industries, which cause serious environmental pollution [45]. Bioremediation of chemicals from the textile industry through vetiver has been reported in various studies. The floating platform technique using vetiver grass can effectively remove 77%, 36%, 88%, and 43% of BOD, COD, SS, and color, respectively, from the textile effluent [46].

#### 3.1.5. Vetiver for Bioremediation in Mining Industries

Mining is an unavoidable task to achieve the inventory demand for mineral needs in developed and developing countries. Mining operations generally cause huge scars in the land, forming large soil dumps containing several heavy metals and completely abrogating the possibility to use that land and its surrounding areas for agriculture or other applications due to mineral runoff [47,48]. Vetiver has the capability of removing heavy metals and production of oil in contaminated mining soil [49]. Vetiver cultivation in such dump soil decreases the contamination level, showing soil-erosion control and mineral absorption capacity. Vetiver can remove several heavy metals like Al, Cr, Cu, Fe, Mn, Ni, and Zn from iron ore soil. It can also remove lead from contaminated soil [50].

#### 3.1.6. Vetiver for Bioremediation in Nuclear Industries

Radioactive compounds generated from nuclear fuel mills, nuclear power plants, and/or during nuclear weapons testing may enter the food chain and lead to health hazards. Vetiver cultivation may be helpful in the bioremediation of nuclear waste [51,52]. Vetiver minimizes soils contamination by stabilizing uranium and thus reduces its movement to the food chain [53]. Vetiver plants grown individually in ^90^strontium and ^137^cesium solution removed around 94% and 61% of their amount, respectively, after a period of 168 h. The plant grown in a mixture of ^90^strontium and ^137^cesium reduced these radioactive compounds to 91% and 59%, respectively [53]. Vetiver has also been reported to treat cesium- and uranium-contaminated soil [54].

#### 3.1.7. Soil Stabilization and Residual Water Phytoremediation

As vetiver grass is capable to adapt itself to extreme environments, even to drought or long rainy periods, it has been explored for slope stabilization. Slope stabilization is fundamental in urban areas and highways as a life protection measure. The use of vetiver grass for soil stabilization has been known worldwide as the Vetiver System. Besides this slope stabilization utility, vetiver grass has also been used for marginal area drainage [55,56]. Degraded area remediation is a major challenge for maintaining productive lands both in urban environments and rural areas. Physical–chemical soil remediation methods are often expensive and may result in the soil ecosystem deterioration. Therefore, soil stabilization, soil treatment, and protection against erosion are alternatives to Vetiver Systems that could complement or even replace traditional engineering techniques [57].

The Vetiver System mechanism uses the plant’s capability to adapt to different environments. Regarding the case of slopes, vetiver grass barriers are placed through the slope surface, keeping a 15 cm distance between each planted unit. Eco-physiological studies have shown that these grass roots may grow from 15 to 20 cm in just 90 days. Even though the root growth rate is reduced after this period, they control the speed of water flow on the surface of the land, since it is still in the penetration and mooring phase in the resident soil. The strong and deep roots (2 to 6 m deep) also help stabilize the soil by preventing landslides. The enveloping action of vegetation roots can significantly reduce both the rate of expansion and the soil’s expansive force. The greater the vetiver root content in the soil, the less compacted the expansive soil becomes [58].

Vetiver is also highlighted in wastewater treatment. Wastewater coming from mining and agricultural activities may lead to toxic agents and heavy metal accumulation due to the use of pesticides, foundries, and refineries. The removal of both metallic and organic contaminants takes place through the roots. The roots absorb Cd^2+^, Cu^2+^, Fe^2+^, and Zn^2+^ ions, which are later incorporated into the metabolism and transported to the plant’s shoots [59,60]. Vetiver is used in wastewater from cardboard mills to remove 66% of lead and 64% of cadmium [61].

In this mechanism, metal ions are transported through the roots’ xylem and phloem vessels, which leads to an accumulation of these metals in the leaves’ cellular vacuoles. Under flooding conditions, it must be highlighted that the irrigation effects using saline water led to a higher phosphate concentration in the vetiver roots. This causes known oxygen-reducing water stress that decreases phosphorus absorption [55]. The absorption capacity of metal ions and other contaminants is observed in the composition of metabolites obtained from the plant. This potential is deeply related to bioactive compound extraction, mainly due to the full harness of biomass generated by the grass in other industrial sectors [62].

#### 3.1.8. Biorefineries Applied to the Production of Vetiver Derivatives

The industrial sector is increasingly looking to optimize production cycle processes to make them more efficient and consequently more profitable. Table 1 shows the main applications of vetiver derivatives from biorefineries. Their leaves and roots have a crucial role as essential oil-extracting sources, which remains the main target of chemical industries [63]. In addition, there are other refined products that may be obtained from the root after the extraction of the essential oil. Perfumes and cosmetics with high added value are examples of these products. Some of them are used by the most famous brands on the market, such as Chanel No-19 by Chanel, Pure Vetiver by Azzaro and Guerlain’s Vetiver Men’s Perfume [64].

Useful pulp for paper production can be obtained from vetiver leaf processing due to its high cellulose content. Cellulose production employing vetiver grass and aqueous mixtures of NH_4_OH and KOH enables the production of printing paper [64]. Textile fibers are another example of products that have been manufactured on an experimental scale using vetiver leaves, which shows the growing interest in this raw material from several industries, such as the automotive industry. Recent studies concerning these fibers conclude that they present good mechanical strength, especially if they are hybrid-produced. Vetiver fibers associated with epoxy, vinyl ester, or polyethylene are some examples [65,66].

In addition to paper pulp and textile fiber, vetiver leaves also yield two derivatives: furfural and biofertilizers. Furfural is a key chemical compound for both isolation and synthesis industries [67,76]. Biofertilizers are incorporated into different mechanisms within the agro-industrial sector. The first process that must be mentioned is the isolation of substances from leaves, which act as fertilizers for crops, such as beans. Other mechanisms use processed leaf residues as biofertilizers through biochar [68].

Vetiver root may be useful in two different ways. The most important one is essential oil extraction. Refined products are derived from this oil and most of them are of interest to both pharmaceutical and fragrance industries. The second root usage option is its role as waste. Besides their application as fertilizers, they are also used in activated carbon manufacture. These alternatives contribute to a more efficient production cycle for vetiver, reducing potentially harmful waste to be disposed in the environment [72,73,74,75,76].

#### 3.1.9. Patents Generation from Vetiver Plant Derivatives

##### Scope for Patents Using Vetiver

Novelty, innovative steps, and commercial applications are the three salient features for a patent [77]. The essential oil from vetiver (VEO) has a complex composition, and a variety of chemical structures of its constituents are responsible for its versatile biological activities. Simple chemical modifications of its compounds can profoundly affect applications, and numerous additional derivatives can be prepared [78]. These derivatives may have different efficacy, as well as different biochemical targets. The biological activity of VEO is likely due to the synergistic action of different compounds present in it. The use of the alcoholic extract of vetiver roots and the simple modification of its certain functional groups (e.g., -CH_2_-OH to -CHO/-COOH, -CHO to -COOH or -CH_2_-OH, =CO to -CHOH, -OH to -OCH_3_, etc.) present a wide scope for in vivo studies on patentable products.

##### Look for Symbiotic Association from Vetiver Mycorrhiza to Other Green Plants

Adams et al. have reported that the vetiver essential oil content is 17.5 times higher in the normal (non-cleansed) roots of vetiver compared to the cultured (cleansed) plant. Gas chromatography–mass spectrometry (GC-MS) analytical data revealed that the tissue-cultured (cleansed) vetiver roots produce large amounts of C19–C29 alkanes and alkanols [79]. These chemical classes in the cleansed plant reduce the concentrations of the typical vetiver oil components. Furthermore, they have quoted that an unidentified biotic factor appears to enhance oil production in normal vetiver, resulting in the generation of signature oil compounds. The oil obtained from the cleansed vetiver has a very different aroma compared to oil from normal vetiver oil [79]. There is scant information in studies regarding the use of mycorrhiza to increase the essential oil content and aroma in *Cymbopogon*, *Lavandula*, and even high-value localized fruits of *Capsicum chinense*. The patented technology, if any, suggested above, will have a marked impact on the socio-economic aspects.

##### Developing the Organic Antibacterial Formulation

The activity of the essential oil recovered from the roots of vetiver has allelopathic effects on certain bacterial organisms, especially belonging to the Enterobacteriaceae family. Srivastava et al. reported that essential oil extracted from both types of roots (infected and non-infected with mycorrhiza) exhibited different effects on the microbial population. The use of oil from plant material with the mycorrhizal association has quite a different response on the bacteria isolated from experimental pond water systems compared to oil from a plant without a mycorrhizal association. However, more research is needed on the bacterial population belonging to other families [80].

##### Nutraceutical from Vetiver as a Synergistic Combination

Glory Josephine et al. reported that ethanolic extract has anti-depressant-like activities, and the combination of fluoxetine (10 mg/kg) with vetiver (100 mg/kg) is effective in reducing forced swim test (FST)- and tail suspension test (TST)-induced depressive behaviors in rodent models compared to the control, though its neurochemical mechanism is not clear [81].

##### Formulation Based on Synergism for Cancer Treatment

Vetiver essential oil (VEO) at 100 ppm in cancer cell lines has been reported to inhibit the growth of 89% of SiHa cervical cells, 88% of CaSki cervical cells, and 89% of MCF-7 breast cancer cells [82]. There are promising opportunities to look at its synergistic formulation with different herbs, e.g., hydro-alcoholic extract of *Catharanthus roseus* for the treatment of different cancers.

##### Herbal-Based Drug to Overcome Various Drug-Resistant Bacteria

VEO is used in medicine and perfumery [83]. It is highly effective in killing drug-resistant bacterial strains, particularly strains resistant to the quinolone and fluoroquinolone class of antibiotics. Because the mechanism of action of all quinolones and fluoroquinolones against bacteria is similar, the development of resistance to one antibiotic would confer simultaneous cross-resistance to other quinolones and fluoroquinolone drugs [84]. Hence, VEO has broad potential in investigations as an herbal-based drug to overcome various drug-resistant bacteria. Currently, vetiver essential oil is increasingly popular in products for topical uses along with aromatherapy [79].

### 3.2. Phytochemical Studies Aimed at Prospecting Biomolecules

#### 3.2.1. Main Phytochemical Differences Between Commercial Vetiver Essential Oils

**Figure 1:**
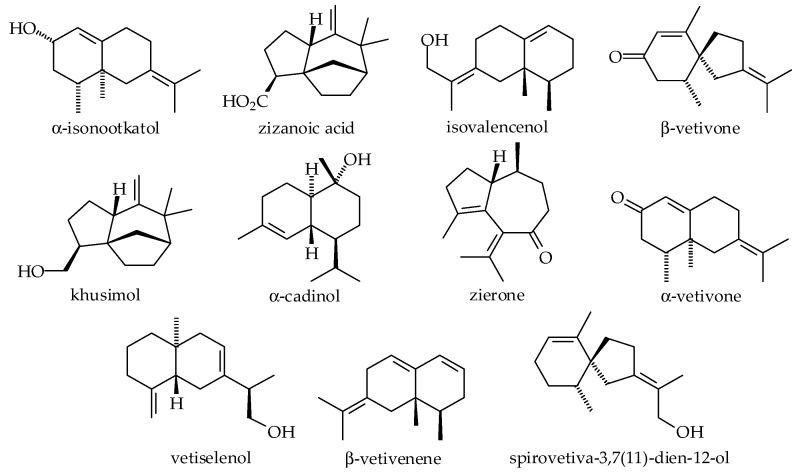
Chemical structures of the most concentrated secondary metabolites present in the *V. zizanioides* Haitian species.

The importance of VEO for the industrial sector is due to its diverse chemical composition. VEO presents high concentrations of sesquiterpene alcohol content, and the most concentrated alcoholic molecules found within VEO are khusimol, isovalenenol, and vetiselinenol (Figure 1). There are also other alcohols present, which may be classified as either primary, secondary, or tertiary alcohols. Phytochemical studies focusing on the most commercialized vetiver variants have attested that globally distributed VEOs’ chemical compositions show little variability [85].

Table 2 shows the main constituents (bold numbers as the main compounds) of each of the 20 commercial vetiver essential oils from Madagascar, India, the Philippines, Ghana, Guatemala, Haiti, and Indonesia based on GC-MS data supplied by the Aromatic Plant Research Centre (APRC) (unpublished data from the authors).

A study conducted with VEO from nine different countries (Brazil, Haiti, China, India, Java, Madagascar, Mexico, Réunion, and El Salvador) showed that their compositions were considerably homogenous. The main observed difference was concerning the oxygenated sesquiterpene khusimol content, which presented an average variation from 12.6% ± 1.1% to 6.1% ± 1.9% among the EOs from these countries [86]. Table 2 shows a notable shift in its percentage as compared to nations like Indonesia, Guatemala, and the Philippines.

**Figure 2:**
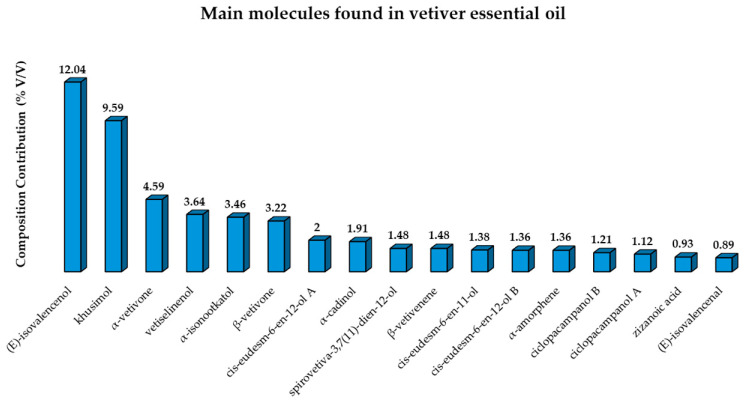
Major components found in vetiver essential oil and their percentages.

Chemical characterization studies described the metabolic profile of Haitian VEO, as it is the most commercialized worldwide. These studies applied chromatographic techniques associated with professional olfactory evaluation. The most highly concentrated secondary metabolites within the Haitian species (Figure 1) that are directly responsible for the EO odorous properties are khusimol, β-vetivone, and α -vetivone. The major components found in vetiver essential oil and their composition contribution are described in Figure 2 [87,88,89].

VEO fractionation methodologies with open column chromatography were also used in phytochemical studies to obtain Haitian VEO fractions. These studies provided a more precise view of the components responsible for the characteristic vegetable odor. The characterization of these substances is extremely important to highlight VEO quality and safety. This plant derivative dereplication allows industries to convert bioactive molecules into bioproducts through biotechnology.

Due to their great chemical complexity, which includes about 200 compounds already listed in the literature, vetiver oils have long been a challenge for analytical chemists. The majority of them are derivatives of sesquiterpenes, with minor components such as acids, alcohols, aldehydes, ketones, and hydrocarbons. When investigating complex essential oils like vetiver oil, it has been necessary to fractionate the entire oil in order to evaluate its fractions and determine a more exact chemical makeup. This method involves evaluating complex combinations with hundreds of structurally linked elements [90].

VEO’s high chemical diversity highlights the need to use new technologies to isolate compounds of interest to the industry. The technological processes used, despite their increasing robustness, are still incapable of guaranteeing efficiency in either the isolation or yield of bioactive compounds. This leads to the usage of relatively crude VEO product formulations. The use of biotechnology has brought new horizons to industries regarding the use of isolated high-added-value molecules extracted from vegetal matrices such as vetiver.

**Figure 3:**
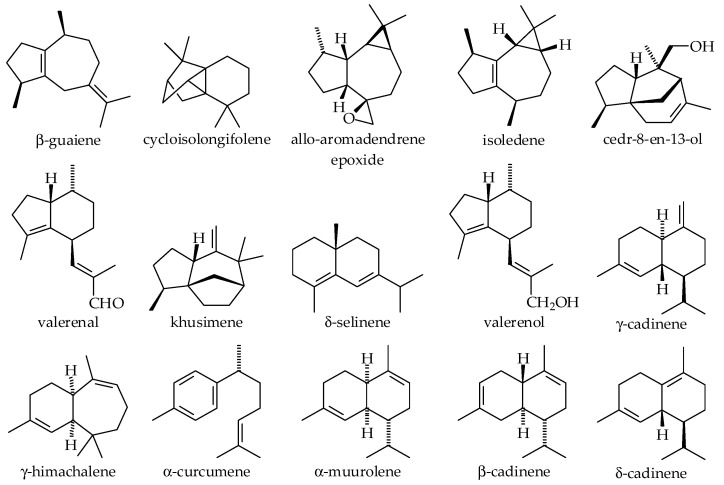
Primary isolated compounds from *Vetiveria zizanioides* essential oil.

This plant species became a commercially competitive product in chemical and pharmaceutical scenarios. Various extraction methods have been utilized to understand and isolate chemical compositions available in vetiver plants. It has been reported that hydrocarbons, carbonyl compounds, alcohol, and carboxylic acids are relatively abundant in vetiver oils [79,90]. Although many compounds from vetiver have been isolated and studied, the majority are demonstrated in Figure 3 [79,88,91,92,93,94,95,96,97].

#### 3.2.2. Technologies Used in Scale-Up Essential Oil Biomolecules Obtention

VEO is an economically important plant derivative due to its versatility in bioactive compounds. The plant is cultivated for its fragrant roots, which yield vetiver oil, an essential oil. This oil is mostly employed in elite perfumery, where its enduring scent makes it a valuable fixative when combined with other fragrances [86]. The cells of vetiver plants are covered with a cell wall of cellulose and pectin, as any plant organism, representing a hard barrier to overcome through the most conventional extraction methods, such as indirect vapor distillation (IVDF) and hydro distillation (HD).

One of the most widely applied extraction methods to obtain high value-added vetiver by-products is supercritical fluid extraction (SFE). Besides its effectiveness, this technique is of the industry’s great interest due to its sustainable aspect and its green chemistry relation. It omits the use of organic solvents, leading to a reduction in chemical waste generation, which is potentially harmful to the environment [83]. Carbon dioxide (CO_2_) is among the most viable solvents for this application due to its low viscosity and high solubilizing power when compared to other applicable gasses.

Phytochemical investigations found that the VEO cultivated in Brazil returned different results (both qualitatively and quantitatively) depending on the extraction method used [83]. Two extraction methods (HD and supercritical fluid with CO_2_) were compared in two different scenarios, either with or without enzymatic pretreatment. The results obtained demonstrated that enzymatic pretreatment improved the EO yield. As for the extraction efficiency, the SFE method showed the best result when evaluating time and yield parameters. It produced a 3.2% extraction yield in a significantly shorter time than the others. Extraction with carbon dioxide-expanded ethanol—CXE is another alternative to EO extraction reported in recent studies. The results obtained through this green method were compared to those acquired using traditional methods (HD and indirect steam distillation) as well as those established by SFE. The analysis showed that the total extraction yield (the amount of oil obtained divided by the amount of original sample) was higher for CXE (7.42%), followed by SFE (~5%), HD (0.6%) and IVD (0.3–0.5%).

Given the obtained data, it was also possible to correlate the antioxidative activity of VEO depending on the extraction method used. According to one study [3], vetiver extract obtained using CXE exhibited two significant properties: antibacterial and antioxidant activity. The DPPH free radical scanning assay was used to assess antioxidant activity, and the median inhibitory concentration (IC_50_) values ranged from 1.39 to 4.54 mg/mL. For the oils’ antioxidant activity, the following order is crucial: supercritical fluid-extracted (SFE) oil (4.54 mg/mL) > carbon dioxide-expanded ethanol (CXE) oil (3.71 mg/mL) > HD (3.57 mg/mL) > IVD (2.19 mg/mL). The analysis established that the VEO showed better anti-radical performance using each extraction method in the following order: IVD > HD > CXE > SFE. This result may be correlated with the EO chemical profile.

**Figure 4:**
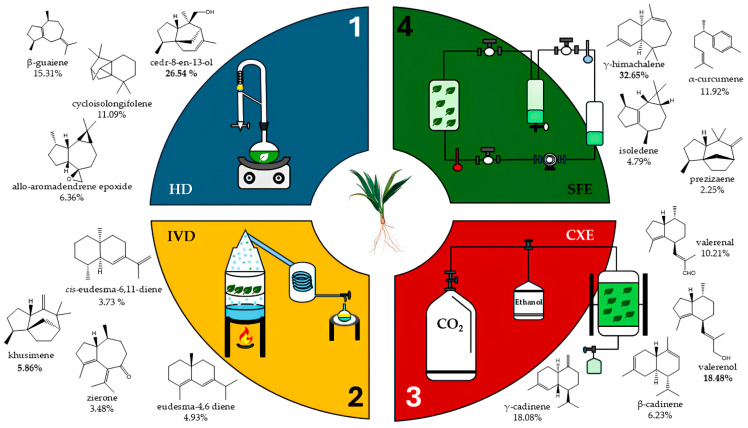
Main compounds and their respective relative yields, obtained from the essential oil of vetiver according to the employed extraction method, adapted from [90,91,92]. ^1^ Vetiver essential oil obtained using conventional hydro distillation (HD); ^2^ vetiver oil obtained using Indirect Vapor Distillation (IVD); ^3^ vetiver grass oil extracted using carbon dioxide-expanded ethanol extraction (CXE); ^4^ vetiver oil obtained using supercritical fluid extraction (SFE).

Chromatographic characterization of the rather diverse range of chemical compounds found in CXE revealed that their relative concentrations vary based on the extraction technique employed, which can have a direct impact on biological activities [88]. The dependence on which extraction method is used influences not only the CXE yield, but also the chemical compound concentrations. In the HD and IVD cases, cedr-8-en-13-ol was the major compound present in the extracted yield using CXE (26.54% and 9.74%, respectively). In the CXE case, valerenol was identified as a biomarker, with a relative amount of 18.48% and, in the SFE case, the most abundant compound was γ-himachalene (32.65%). γ-Himachalene was the compound with the highest relative yield among all the molecules characterized in extraction using CXE through different methods, analyzed with GC-MS (Figure 4).

The variations between the molecules obtained using different extraction methods indicate that to optimize the CXE biomolecule yield, the extractive process must be taken into account. A significant number of these compounds are obtained from chemical interaction with other molecules present in the CXE. Depending on the specific conditions of each extraction method, precursor molecules may react either between each other or undergo decomposition reactions. Even interactions with the extracting solvent may influence the results, so as to present different chemical profiles [94].

### 3.3. Vetiver Plant Derivatives and Their Potential Pharmacological Activities

#### 3.3.1. Studies on the Biological Activities of Vetiver Essential Oil

Traditionally, vetiver has been utilized in India, Indonesia, Pakistan, Senegal, Sri Lanka, Thailand, Mauritius, and Nigeria for various medicinal and aromatic benefits [95]. In traditional medicine, vetiver has been utilized for various ailments such as mouth ulcers, boils, epilepsy, burns, snake bite, scorpion stings, rheumatism, lumbago, sprains, fever, headache, cardiac debility, palpitation, fainting, emotional stress, urinary tract infections and calculi, disease of gall bladder, inflammation, polydipsia in children, vomiting in cholera, and irritability of the stomach [92,96]. Furthermore, vetiver has been used as an abortifacient, antiseptic, anthelmintic, stimulant, diaphoretic, refrigerant, tonic, aphrodisiac, etc. Also, vetiver oil has been considered for its regenerating, skin cooling, and body odor abolishment effects [97,98,99,100,101,102,103,104,105,106].

**Figure 5:**
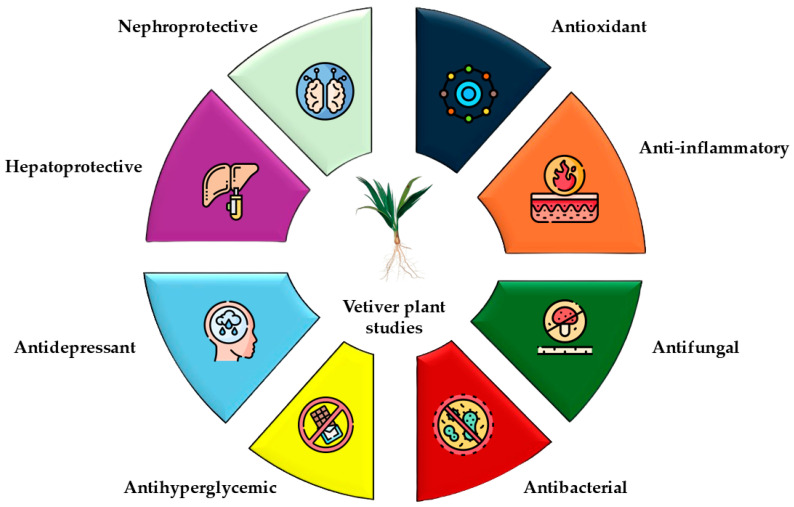
Different studies where vetiver plant and its derivatives are used.

The vetiver plant and its derivatives have been studied for many pharmacological activities, such as antifungal, antibacterial, antitubercular, antihyperglycemic, antidepressant, hepatoprotective [107], antioxidants [108], anti-inflammatory [73], and nephroprotective [109] (Figure 5). The essential oil of vetiver root has been shown to possess antioxidant activity. A significant protective effect of root extract was also observed in reduced glutathione and malondialdehyde concentrations of erythrocytes subjected to oxidative stress by *tert*-butyl hydroperoxide and hydrogen peroxide [110,111]. Vetiver essential oil (VEO) possesses strong free-radical-scavenging activity when compared to standard antioxidants. Among the complex constituents in crude vetiver oil, β-vetivenene, β-vetivone, and α-vetivone, which have shown strong antioxidant activities, were isolated and identified using various chromatographic techniques including silica gel open-column chromatography, silica HPLC, and GC-MS [112]. Crude VEO and some of its inherent antioxidant constituents could be considered as novel natural antioxidants, which might have alternative potential applications. Moreover, if a large quantity of α-vetivone and β-vetivone can be easily separated from crude VEO using chromatographic techniques or cost effectively synthesized, it is believed that vetivones will acquire more practical applications in other areas besides their current utilization as effective termiticides [113] and cosmeceuticals.

In a study with vetiver essential oil (VEO) from *V. zizanioides*, antinociceptive and anti-inflammatory properties were analyzed [114]. The primary constituents were determined to be khusimol (19.57%), *E*-isovalencenol (13.24%), α-vetivone (5.25%), β-vetivone (4.87%), and hydroxyvalencene (4.64%) using GC-MS characterization. A certain dosage of *V. zizanioides* (L) essential oil demonstrated anti-inflammatory properties. It is primarily used to reduce inflammation in the neurological and circulatory systems. Inflammations brought on by sunstroke, dehydration, and dry winds can also be effectively treated with it.

In different research with VEO, which is used to treat rheumatism and joint and muscular discomfort, VEO showed anti-inflammatory and antioxidant properties [115]. It is a component of formulations for musculoskeletal pain relief and enhances blood flow and tissue oxygenation. As the body’s reaction to wounds and irritations, its anti-inflammatory properties aid in pain relief and inflammation reduction. VEO is frequently used to treat neurological and circulatory system inflammation. It also works well to reduce inflammation brought on by dry winds, heatstroke, and dehydration. It is a natural alternative for treating chronic inflammatory disorders because of its relaxing and rejuvenating effects, which reduce many forms of inflammation [116].

The majority of the studies regarding pharmacological activities have been performed on different extracts of vetiver. However, very few studies have been performed for finding the pharmacological effects of individual compounds isolated from vetiver. Future studies should focus on individual compounds, which should be assessed for important pharmacological activities to establish the full potential of vetiver plant species and their derivatives.

The effectiveness of a natural repellent made of VEO and β-caryophyllene oxide (BCO) against disease-carrying mosquitoes was assessed in a study by Nararak and partners [117]. The mosquito-repellent activity showed escape rates for *Anopheles minimus* ranging from 74.07% to 78.18% and for *Culex quinquefasciatus* ranging from 55.36% to 83.64%; the mixture in a 1:2 ratio demonstrated high efficacy. The combination produced the strongest synergistic impact of BCO + VO (1:2) and showed effectiveness against *Aedes aegypti* and *A. albopictus*, albeit precise rates were not provided for these species. *A. aegypti* did not exhibit knockdown action, while *A. minimus* and *C. quinquefasciatus* did (escape rates 18.18% to 33.33%). The results are encouraging, particularly in terms of controlling the vectors of filariasis and malaria, demonstrating the ability to repel mosquitoes and temporarily incapacitate them. The lack of a knockdown impact on *A. aegypti, however,* indicates that the formulation needs to be modified to increase its effectiveness against this vector. The study also highlights how natural repellents are safer than synthetic ones because they are less harmful to both people and the environment. As a result, BCO and VEO together present a viable and successful vector management option in endemic regions.

Nootkatone, a component of VEO, showed antifeedant and repellent properties against the termite *Coptotermes formosanus*, which is known to harm trees, crops, and lumber. Both were effective at concentrations of 5 µg/g (VEO) and 25 µg/g (nootkatone), which dramatically decreased termite tunneling and paper consumption [118]. Using Tim-bor^®^ (disodium octaborate tetrahydrate) as the reference pesticide, Mao and colleagues assessed the phytotoxicity of these compounds on *Citrus* and *Pisum sativum* (pea) due to the observed pesticidal activity. The findings showed that, in comparison to Tim-bor^®^, nootkatone and VEO inhibited pea development less (nootkatone < VEO < Tim-bor^®^). Furthermore, VEO and nootkatone did not exhibit substantial toxicity, whereas Tim-bor^®^ (2000 μg/g soil) caused up to 38.9% mortality in peas. Citrus tree growth remained unaffected. In a different investigation, Zhu and colleagues assessed nootkatone’s toxicity and repellent efficacy against *Coptotermes formosanus*. Nootkatone demonstrated potent termite-repellent activity and harmful effects even at the lowest tested dose (10 μg/g). According to the research, nootkatone and VEO can be employed as efficient herbicides and pesticides without affecting target plants’ ability to thrive [119].

Despite having no notable cosmetic qualities other than scent, vetiver essential oil possesses exceptional antibacterial activity [120]. In addition to being a powerful inhibitor of *Candida glabrata* and a moderate inhibitor of *Candida albicans*, it has shown effectiveness against Gram-positive bacteria, including *Corynebacterium striatum* and *Staphylococcus aureus* (including methicillin-resistant strains). Fewer side effects and lower treatment costs underscore its potential as a substitute for synthetic biocides. When coupled with cinnamon (*Cinnamomum verum*) essential oil, VEO demonstrated potent antibacterial activity, particularly against microorganisms that cause acne [121,122]. With minimum inhibitory concentration (MIC) values ranging from 0.19 to 0.25 mg/mL, the combination showed promise for suppressing microbes in small quantities. VEO has also been found to provide antifungal effect against *Aspergillus fumigatus*, *Microsporum canis*, *Trichophyton interdigitale*, *T. mentagrophytes*, *T. rubrum*, *C. albicans*, *A. niger*, and *A. clavatus* [123]. In addition, VEO has shown antiparasitic activity against *Trichomonas vaginalis* [124], and nematocidal activity against *Meloidogyne incognita* [125]. In addition to having moderate antibacterial activity against *Bacillus subtilis* (MIC = 312.5 µg/mL), *Pseudomonas aeruginosa* (MIC = 312.5 µg/mL), and *Escherichia coli* (MIC = 312.5 µg/mL), the essential oil extracted from vetiver root using HD had strong antimicrobial activity against *Staphylococcus aureus* (MIC = 39 µg/mL) [3]. The findings indicate that traditional and conventional treatments have a strong antibacterial effect on Gram-positive bacteria, particularly *Staphylococcus aureus*.

In 2020, Atif et al. assessed how well essential oils from *Ocimum basilicum* and *Vetiveria zizanioides* protected jack fruits (*Artocarpus heterophyllus*) from bacteria and fungi that cause spoiling. With MIC values of 75 and 50 μg/mL, respectively, VEO demonstrated strong efficacy against *Proteus mirabilis* and *S. aureus*. EOs from these sources effectively inhibited the mycelia growth of selected fungi at the concentration of 12.5 μg/mL. Additionally, the growth of *Streptococcus mutans*, *P. mirabilis*, *Enterobacter aerogenes*, *S. aureus*, *Penicillium notatum*, and *Rhizopus microsporus* was inhibited by the synergistic impact of *V. zizanioides* and *O. basilicum* [126,127].

Additionally, VEO decreased the growth of *P. notatum* on the jack fruit’s surface, which is mostly responsible for the fruit’s degeneration and spoiling [128]. Dubey and colleagues investigated the antifungal activity of essential oils isolated from Indian vetiver from two different geographical locations (North and South India) against phytopathogenic fungus *Rhizoctonia solani*. Each type of oil showed antifungal activity against *R. solani* in a dose-dependent manner; in particular, essential oil from the South India variety exhibited better fungicidal activity compared to the North India variety. Khusimol is the primary component of both essential oils; in North India, it contributes to up 16.25% while in South India, it makes up 15.77%. In another study, essential oil from the roots of vetiver showed potent activity against four tested wood rot fungi, namely, *Coniophora puteana*, *Gloeophyllum trabeum*, *Coriolus versicolor*, and *Poria placenta* with an MIC value of 200 μL/L [129].

VEO has been shown to provide benefits for skin problems. VEO rejuvenates skin, relieves acne, normalizes oily skin, moisturizes dry skin, and provides soothing effects on skin. VEO reduces tyrosinase enzyme activity, which results in the inhibition of melanogenesis and skin pigmentation. Furthermore, vetiver oil decreases lipid peroxidation and enhances endogenous antioxidants, as indicated by decreased malondialdehyde and increased superoxide dismutase, catalase, and glutathione peroxidase [130].

According to the results of recent studies, vetiver oil shows promise as a medicinal ingredient for treating bacterial and fungal infections with its repellent properties, as well as for use in skin-care cosmetic formulations. Its applicability as a safe and efficient substitute for antibiotic treatments is further supported by scientific confirmation.

#### 3.3.2. Biological Activities of Vetiver Extracts and Components

In reference to vetiver extracts specifically, according to in vitro research, vetiver oil’s khusenic acid and khusimol were superior to conventional medications in their ability to combat several drug-resistant mutations of acid-fast *Mycobacterium smegmatis* bacterial species [131]. They also worked well against *M. tuberculosis* strains. These chemicals have an advantage over the current synthetic medications since they are edible, fragrant natural molecules. The ethanol and hexane extracts of dried and undamaged vetiver (*V. zizanioides*) roots exhibit potent antimycobacterial activity against *M. tuberculosis* H(37)Rv and H(37)Ra strains, according to in vitro research carried out in India [132]. Ethanol extract revealed significant activity at 500 μg/mL as a minimal dose, with 50 μg/mL for the hexane extract. These findings lend credence to vetiver’s long-standing anti-tuberculosis properties [95].

Using the agar well diffusion method, the antibacterial activity of vetiver extracts was assessed against four fungal and nine bacterial clinical isolates. Maximum activity was demonstrated by the methanol extract of vetiver, specifically against *Salmonella paratyphi* (13.9 ± 0.08 mm) and *S. aureus* (15 ± 0.12 mm) [133]. The antibacterial activity of solvent extracts was confirmed by their inhibition of *S. aureus*, *E. coli*, and *P. aeruginosa*. While the methanol extracts of vetiver grass were efficient against *Rhizopus nigricans* and *Candida albicans*, the diethyl ether extracts inhibited *Salmonella typhi* and *Klebsiella* sp. However, *Aspergillus* sp., *A. niger*, and another strain of *Klebsiella* sp. were unaffected by any of the extracts. Aside from a minor inhibition against *E. coli*, aqueous extracts exhibited very little activity [134]. The most vulnerable Gram-positive bacterium was *S. aureus*, which showed inhibitory zones of 15 ± 0.12 mm for vetiver ethanolic extract. The most sensitive Gram-negative bacterium was *Salmonella paratyphi*, which displayed an inhibitory zone of 13.9 ± 0.08 mm in the vetiver ethanolic extract.

Implementing alternative methodologies, carbon dioxide-expanded ethanol extraction (CXE) of essential oil isolated from the roots of *Vetiveria zizanioides* demonstrated weak antimicrobial activity against *B. subtilis* (MIC = 312.5 µg/mL), *E. coli* (MIC = 312.5 µg/mL), and *P. aeruginosa* (MIC = 2500 µg/mL), but relatively strong antimicrobial ability against *S. aureus* (MIC = 78 µg/mL) [3]. Supercritical fluid extraction (SFE) vetiver oil demonstrated a moderate level of activity against the same Gram-positive bacteria, *S. aureus*. Like CXE oil, it was not very effective against *B. subtilis* (MIC = 156 µg/mL), *P. aeruginosa* (MIC = 312.5 µg/mL), or *E. coli* (MIC = 625 µg/mL). This oil shows efficient bacteriostatic and bacteriocide properties, thereby helping cure sepsis. Because of its safety, this oil can be applied externally or administered orally to protect wounds as well as internal organs from sepsis [114].

The root extract of vetiver showed significant antidiabetic activity in the second and fourth hour after administration compared to diabetic control, with a result that was comparable with standard glibenclamide. The study indicates that the ethanolic extract of vetiver root possesses better antihyperglycemic activity than any other extract, in both normal and allaxon-induced diabetic rats [135]. In another study, the methanolic extract of vetiver showed good glycaemic control, antioxidant and hypolipidemic properties, and protection against liver and kidney injury in STZ-induced diabetic rats. It is suggested to be effective for reducing oxidative stress and free radical-related diseases including diabetes [136].

Various brain-related conditions have been reported to be alleviated with the use of vetiver essential oil. It is suggested that vetiver oil can provide benefits in anxiety, stress, seizure, tremor, migraine, ADHD, shock, afflictions, fear, Parkinson’s disease, etc.; because of its sedative properties, it has been suggested to benefit insomnia patients and to sedate those with nervous irritations, afflictions, and emotional outbursts such as restlessness, anger, nervousness, and hysteric attacks [137,138]. The anti-convulsion mechanism perhaps acts through γ-aminobutyric acid (GABA). GABA is an inhibitory neurotransmitter that prevents convulsion [139].

Gupta and colleagues (2013) have shown the anti-convulsion effect of ethanolic extract of vetiver to be comparable to phenobarbital. Furthermore, the GABA-potentiating effect of vetiver may be responsible for providing relaxation and sleep and has been used in jetlag [84]. Vetiver has been reported to reduce depression (Hamilton Depression Rating Scale; HAM-D) as well as anxiety (Hamilton Anxiety Rating Scale; HAM-A) [140]. The ethanolic extract of vetiver showed antidepressant activity in tail suspension and forced swim test-induced depressive behavior [20,78]. Also, vetiver oil reduced levels of cortisol, which is a stress hormone. Children with attention deficit hyperactivity disorder (ADHD) who are hyperactive have been treated with vetiver essential oil. Apathy, desperation, disconnection, disarray, desire to flee, crisis-like emotional states, and distress can all be effectively managed [140]. Additionally, it has been shown to lessen anxious behavior through elevated expression of the c-Fos protein in the central amygdaloidal nucleus’s lateral division, which is important in mood and emotion regulation [141]. The relaxing and anti-anxiety effects of VEO infusion are well known. The earthy, woody scent of VEO is used in aromatherapy to ease muscle tension, encourage sleep, and lower stress [142,143].

An investigation has assessed how VEO affects three different cancer cell types: triple-negative breast cancer (TNBC–4T1), luminal breast cancer (T47D), and colon cancer (WiDr). Because the VEO included a lot of β-caryophyllene, a cannabinoid that has a high affinity for the CNR2 receptor, the oil showed more cytotoxicity against TNBC cells (IC_50_ = 60 µg/mL) than it did against colon cells. The findings revealed that WiDr and 4T1 cells had a cell cycle block in the G2/M phase, but T47D cells had an increase in the sub-G1 cell population, indicating apoptosis. Furthermore, WiDr and T47D showed a markedly higher level of reactive oxygen species (ROS) than 4T1. The toxicity of the oil seems to be directly linked to β-caryophyllene’s activation of the CNR2 receptor, which promotes cell death, particularly in aggressive malignancies like TNBC. According to these results, vetiver oil might be useful as a chemo preventive treatment for some cancers [144].

Vetiver was shown to possess hepatoprotective action against ethanol intoxication in rats. Also, the methanolic extract of vetiver reversed CCl4-induced acute liver damage, indicated by the attenuation of CCl_4_-induced, increased ALT, AST, ALP, TBL, DBL, LDH, GGT, MDA, IL-6, IL-1β, and TNF-α levels and decreased GSH and TP levels in CCl_4_ control. The authors suggested that attenuation of TNF-α- and Il-6-mediated pathways may be responsible for benefits in acute liver damage [145].

Jindapunnapat and colleagues investigated the activities of crude root and shoot VEO extracts against *Meloidogyne incognita*, known for causing the southern root-knot [125]. The dried root powder and ethanol extracts of vetiver shoots and roots were immersed in 95% ethanol (10% dry weight plant material/volume ethanol) and exhibited 70% and 40% mortality, respectively, against the second-stage juvenile (known as J2) of the nematode. Additionally, both extracts also showed significant repellent activity against J2; however, no significant mortality or repellent activity was observed in vetiver essential oil. Two major constituents, sesquiterpene acid (3,3,8,8-tetramethyl-tricyclo [5.1.0.0(2,4)] oct-5-ene-5-propanoic acid) and alcohol (nardoeudesmol C), determined using GC-MS analysis, were found to be common in the root extract as well as in the essential oil. The content of the acid was found to be relatively higher in the root extract compared to the essential oil. Overall, the study concluded that the constituents isolated from vetiver may be useful for suppressing nematode populations in the soil.

Bhardwaj et al. evaluated the nematocidal activity of vetiver oil, its polar and nonpolar fractions in petroleum ether and acetone, respectively, against *Meloidogyne incognita* nematode. *M. incognita* is a widely distributed parasite that affects the number of crops, particularly in the subtropical and tropical regions. VEO showed better efficacy against second-stage juveniles *M. incognita*, followed by the acetone and petroleum ether fractions. Moreover, EO showed egg-hatch inhibition (50%) at a concentration of 2 mg/mL, while acetone and petroleum ether showed 50% inhibition at concentrations of 4 and 6 mg/mL, respectively [146].

**Figure 6:**
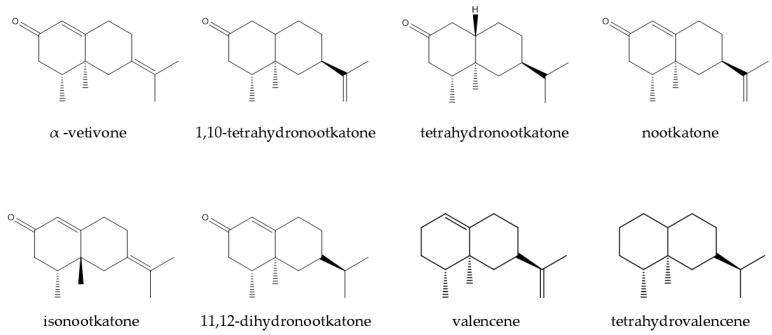
Structure of eight valencenoid derivatives evaluated against Formosan subterranean termites.

In another study, nootkatone, a ketonic sesquiterpene found in VEO, was examined for its ability to resist and kill Formosan subterranean termites (*Coptotermes formosanus*). The findings showed that nootkatone had a potent poisonous and repellent effect on termites, even at the lowest dose tested (10 mg/g of substrate). In a dose-dependent manner, its presence dramatically decreased the consumption of cellulose, while the death rate rose with increasing concentrations, reaching 95% at 200 mg/g. Moreover, termites were unable to dig tunnels at concentrations greater than 20 mg/g, indicating an inhibitory effect on motility. According to the study’s findings, nootkatone may be utilized in wood treatment and agricultural pest management, as well as a natural termite barrier. Eight valencenoid derivatives were evaluated (Figure 6): 1,10-dihydronootkatone, tetrahydronootkatone, isonootkatone (α-vetivone), nootkatone, 11,12-dihydronootkatone, nootkatol, tetrahydrovalencene, and valencene for their repellent activity against Formosan subterranean termites [147].

Bahri and colleagues isolated a phytoconstituent from vetiver grass roots and evaluated its repellent activity against *Cryptotermes* sp. termites. A new isolated compound from the hexane fraction, identified as 2-methyl-1-butylamine or 1-amino-2-methylbutane, possessed potential repellent activity against *Cryptotermes* sp. termites at a concentration of 0.025% [71].

*Tribolium castaneum*, also known as red flour beetle, is well known for the post-harvest damage of grains, particularly wheat [148]. In another study, root extracts of vetiver using different solvent systems, viz. acetone, methanol, ethyl acetate, and petroleum ether were evaluated for insecticidal activity against the four strains of red flour beetle. The petroleum ether extract exhibited the highest toxicity against the larva as well as adult pest with LD_50_ values of 0.051 and 58.69 g/cm^2^, respectively, while methanol and acetone extract showed the lowest toxicity against the larva and adulty pest, respectively [149].

**Figure 7:**
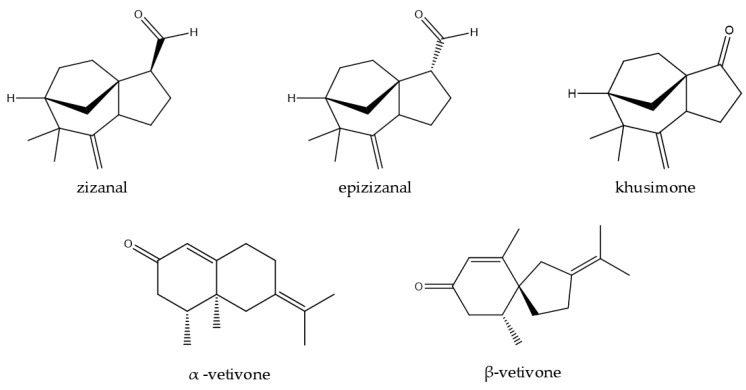
Five phytoconstituents from vetiver oil reported for insect-repellent activity.

Two novel sesquiterpene aldehydes, zizanal and epizyzanal, were discovered during the investigation of insect repellent chemicals found in VEO. These molecules, which showed repellent qualities similar to those of other oil components like α-vetivone, β-vetivone, and khusimone, were discovered after the most active part of the oil was separated and refined. Spectroscopy and chemical synthesis from zizanoic acid were used to confirm their chemical structures. The findings demonstrate the potential of vetiver oil and its components as efficient natural insect control substitutes. The insect-repellent activity of five phytoconstituents isolated from the EO of vetiver or their semisynthetic analogs, namely, zizanal, epizizanal, khusimone, α-vetivone, and β-vetivone (Figure 7), was reported by Jain and his research team [150].

The use of vetiver oil extracts as poisonous agents and repellents against pests is described in Patent US6906108B2 [151]. Nootkatone’s effectiveness was evaluated in comparison to N,N-diethyl-m-toluamide (DEET), a well-known repellent. Nootkatone had both toxicant and repulsive effects in tests with fire ants (*Solenopsis invicta*). After 15 min, the number of insects in the treated area dropped to 23.89% at 10 µg/g and to 13.33% at 100 µg/g. The control group recorded 75.56%. At the same quantities, vetiver oil also showed a repelling effect, reducing to 27.22% and 33.33%, respectively. The control group experienced a cumulative mortality of less than 5% after seven days, but the highest concentrations of vetiver and nootkatone were shown to result in the highest death rates. Additionally, even 40 days after application, these chemicals’ effectiveness persisted. Nootkatone repellence reached 100% after 72 h in tick (*Ixodes scapularis*) testing, although DEET, which was employed as a positive control, occasionally lost its effectiveness after 90 h.

Nootkatone was more effective as an insecticide and repellent than DEET, demonstrating significant repellent and toxic properties against ants, ticks, and cockroaches, as evidenced by its 90% fatality rate after 96 h compared to DEET’s 50%. Nootkatone also showed a substantial repellent effect on cockroaches (*Blattella germanica)*, showing 100% efficacy after an hour at a dose of 10 mg and 70% at 1 mg, whereas DEET only showed 50% repellence. Nootkatone, however, largely functioned as a repellent in this situation, as seen by the fact that none of the tested treatments caused appreciable death. Additionally, nootkatone continued to work for at least 40 days following application. The substances C-cedrene, zizanol, and bicyclovetivenol were also shown to be possible insect repellents in addition to nootkatone. As a natural substitute for synthetic pesticides, nootkatone has been proven to be safe for both people and the environment. According to the patent, these substances may be applied in home and agricultural settings to shield surfaces and materials from pest infestations [152].

The striped stem borer (*Chilo suppressalis*) is known for attacking and damaging rice stems; particularly, its outbreaks have been reported in certain parts of China. A study performed by Yan-hui Lu et al. revealed that a vetiver grass plantation acted as dead-end trap for this pest, which also suppressed the growth of its larvae and overall provided better environmental conditions for the growth of rice [89].

#### 3.3.3. Main Vetiver Vegetable Derivatives Diffused in the Pharmaceutical and Cosmetic Industries

There is a growing demand for the use of more ecologically responsible bioproducts in many industrial sectors. VEOs are important raw materials for numerous modern fragrance formulations. These plant derivatives have been widely used in several countries, especially European countries, which are the global EO market protagonists, followed by the Asia-Pacific region and North America [88].

**Figure 8:**
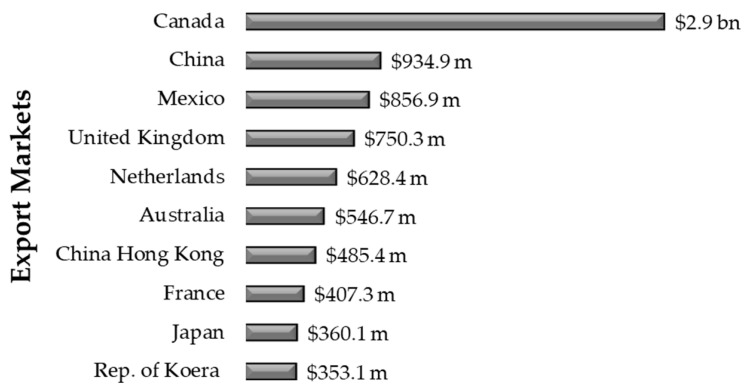
USA—10 export markets for essential oils, perfumes, cosmetics, and health products (2020).

EO exports present another very intense economic scenario to cosmetic and pharmaceutical industries. In 2020, the United States surpassed USD 12.4 billion in plant derivative exports, whose use is growing due to the strong demand for high-purity natural ingredients in bioproduct compositions. According to COMTRADE (United Nations Commodity Trade Statistics Database), Canada was the first country to reach the largest market for natural products. It was responsible for USD 2.9 billion (23.1%) in exports to the United States, followed by China with the USD 934.9 million (7.6%), and Mexico with USD 856.9 million (6.9%). USA imports involved not only essential oils, but also natural product-based perfumes, cosmetics, and sanitary products (Figure 8) [94].

**Figure 9:**
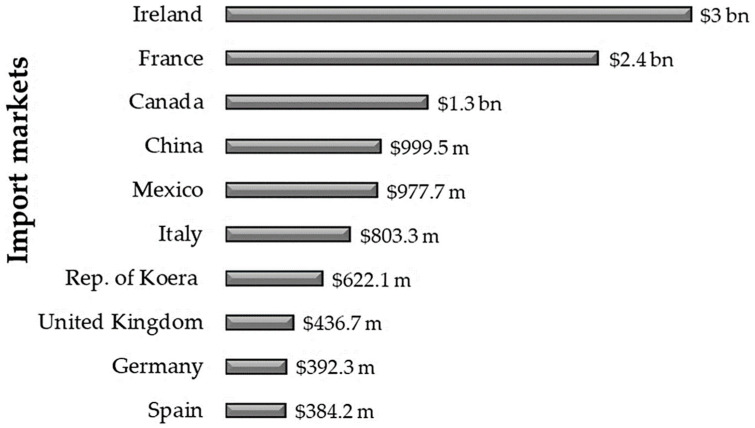
USA—10 import markets for essential oils, perfumes, cosmetics, and health products (2020).

In 2020, regarding these vegetable derivative imports, the United States surpassed the mark of USD 13.6 billion considering the overall calculation of the main markets. The import market analysis showed that Ireland stands out as the largest import market country for the United States. It was responsible for USD 3.0 billion (21.8%) imports, followed by France—USD 2.4 billion (17.5%)—and, finally, Canada as the third largest market—USD 1.3 billion (9.8%)—for essential oils, perfumes, cosmetics, and sanitary products (Figure 9) [153].

VEO demand, which follows the growing vegetable derivative market, comes mainly from the food and beverage (35%), fragrance, cosmetic and aromatherapy (29%), hygiene (16%), and pharmaceutical (15%) segments [149]. These data corroborate the spread of conscious consumption adopted by contemporary generations. It has been notable how healthier practices for both human health and the environment are increasingly in focus. This has led to volatile oil consumption as food sector additives to not only flavor beverages but also to be added in creams and aromatic product formulations. VEO therapeutic properties have become increasingly popular and are currently used as alternative therapies in treating stress and anxiety problems [154].

The global essential oils market has also been affected by growing organic and natural hygiene product demand due to increased consumer awareness about health problems inherent to petroleum derivatives and synthetic chemical compounds, in general. Vetiver root infusion has been used as a tranquilizer, febrifuge, diaphoretic, stimulant, stomach, antispasmodic, astringent, and excellent supplement to improve blood circulation for therapeutic purposes [154]. Its high chemical complexity justifies VEO’s wide employability. The classes of terpenoids and phenylpropanoids also present within VEO are remarkably recognized for their anti-inflammatory, antiseptic, aphrodisiac, healing, and tonic efficacy. These biomolecules are used for bone strengthening, rheumatism treatment, gout, arthritis, muscle pain, and cramps [155]. Vetiver root essential oil (VEO) is used to treat emesis, colic, and flatulence, as well as a potential diuretic in folk medicine. The use of this plant derivative has wide commercial acceptance due to its efficiency and safety in diverse bioavailability models and formulations.

VEO use in food or pharmaceutical industries may have some restrictions due to its aroma, flavor, volatility, and low chemical stability in hydrophilic media, like the most essential oils. This EO requires specific care in its handling, since it is also sensitive to oxidation, heat, humidity, and light. Nanoencapsulation is a technology that looks forward to bypassing these peculiarities. This strategy provides a development formula capable of encapsulating VEO’s bioactive components and increasing its capability to become soluble even in microemulsions and nanoemulsions [156].

The nanoencapsulation of active compounds has two functions. It aims to increase not only oxidative stability but also other properties such as thermostability, photostability, shelf life, and biological activity. Guaranteeing its delivery to a specific target is its second function. EO encapsulation controls volatility, sensory characteristics (mainly odor and taste), and its active ingredient release properties, which ensures both prolonged chemical stability and biological activity under storage conditions. Nanoemulsion systems are characterized by liposomes, which result in the formation of self-assembled vesicular systems. This system is characterized by one or more phospholipidic bilayers that surround an aqueous core. The drugs are disposed in a microsphere form called miscella in this type of vehicle’s vesicular lipid-based delivery formulation [157,158].

Microemulsions are nanostructures that can be formulated within the size range of 100–400 nm, which present greater kinetic stability and better storage stability. Nanoemulsions, on the other hand, are formulated within the range of 1 to 100 nm, and they are characterized by isotropic dispersions of two immiscible liquids (such as oil and water) that undergo emulsification of the oil and water phases using an emulsifying coating compound. An immiscible phase union is only possible due to the presence of a substance capable of reducing the interfacial tension between phases. This kind of substance is called a surfactant [159].

Nanoencapsulation provides controlled active ingredient release, which increases VEO bioavailability and efficiency besides providing a more robust, safe, and efficient pharmacokinetic profile. These characteristics take vetiver’s vegetable derivatives to another industrial level. It is possible to carry out the isolation of target substances of clinical interest through encapsulation technologies. It makes biomolecules present in VEO susceptible to formulations that follow pharmaceutical guidelines regarding the safety, efficacy, and quality of developed bioproducts [160].

### 3.4. Vetiver Essential Oil Biomolecules of Industrial Interest

#### Lignin Prospection from Vetiver Plant Derivatives

Plants’ primary or secondary metabolic phytochemicals are biological defense tools for plants. Lignin is among these chemical compounds and has been studied in different applications through biotechnological tools [161,162]. Lignin is a multi-substituted phenolic polymer and forms 15–30% in weight of dry lignocellulosic material, with cellulose, hemicellulose, and lignin as main components.

The first concepts of biorefineries were communicated in Europe about a decade ago. They looked at plant organisms as true high-complexity factories of active compounds. Lignin is currently studied as one of the most promising candidates for the biorefinery platform, and it can be used for aromatic chemical production and energy supplementation. This vegetable secondary-metabolite-derived compound present in the leaves and roots of vetiver has great prominence within vegetable anabolic pathways [163].

Specific lignins provide significant antilipid peroxidation and oxygen radical-scavenging effects. Significant lignin inhibitory effects can also be observed in both central nervous system and cancer cell proliferation [161,162]. Studies indicate that lignin-based nanocomposites have a promising future in the biomedical field. Lignin-based nanocomposite molecular expression, biocompatibility, and cytotoxicity in cell lines brings the technology that was needed for new material and product development from vetiver leaves and roots using their biomass as a matrix [164].

Biotechnology studies have used lignin in several industrial processes. It has been used as an emulsifying agent in the food industry, as an alternative antioxidant to synthetic antioxidants, as a pesticide on crops, and as a fertilizer in agriculture. Lignin is a raw material used in nanomaterial formulations in cosmetology, such as in active drug or cosmetic nanocapsules. Advances in biotechnological tools have enabled vetiver biomass lignins to gain an increasingly broader application spectrum. These lignins can also absorb wavelengths from the ultraviolet to the visible spectrum in a polychromophoric complex, which represents an innovative usage for this compound. Therefore, while they give dark shades to vegetable fibers, it makes them viable for the development of sunscreens with a significant ability to protect against ultraviolet rays, for example [165].

Studies have demonstrated that it will be increasingly common for oxidative stress reactions to be related to dermal pathologies in the next few years due to the advance of global temperature imbalances and the prolonged exposure of people to ultraviolet light. This sun exposure at high temperatures produces a large amount of radical substances, which are associated with both oxidative damage and cell metabolism disorders. The use of biotechnology for the application of lignin in the development of sun filters is a preventive alternative for this scenario. This is possible due to the excellent antioxidant and UV absorption capacities of this biomolecule [166].

Lignin’s natural polyphenol extract can not only effectively filter out ultraviolet rays but also repair damaged DNA. Lignin’s physical and chemical characteristics (such as low toxicity, resistance to ultraviolet rays, and antibacterial and antioxidant activity) are attracting more and more attention from the pharmaceutical industry [167]. Contemporary technology employed in nanolignin preparation procedures mainly includes anti-solvent precipitation, self-assembly, gradual addition, and mechanical methods, which gives robustness to this technique. It also provides efficiency regarding active ingredient bioavailability [168,169].

## 4. Conclusions

This review presented a vast and important group of applications for *Vetiveria zizanioides*. This plant species of grass has been used in several environmental aspects, either as a bioremediation agent in the prevention of soil erosion, due to its long roots and its resistance to changes in temperature and pH, or for its specialized metabolism, composed of bioactive molecules of great importance in the pharmaceutical industry. Even with the biological and chemical studies already carried out on this species, the biotechnological applications are still in the early stages, showing a timid but promising scenario for this plant to become a major protagonist of the contemporary bioeconomy. Thus, studies aimed at the development of new technologies and innovative bioproducts emerge as the future of a new production chain in the agroindustry, transforming this plant matrix into a source for prospecting high-added-value bioproducts.

## Figures and Tables

**Table 1:** Refined vetiver grass biomass derivatives.

Component	Target	Reference
Leaf	Paper pulp	[64]
	Textile fibers	[65,66]
	Furfural	[64,67]
	Biofertilizer	[68,69]
Root oil	Aroma	[70]
	Termite-repellent terpenoid	[71]
	Anti-hypertensive and anti-spasmodic	[72]
	Anti-inflammatory agent	[73]
	Gum microcapsules with antidepressant activity	[74]
Oil Free Root	Activated charcoal	[75]

**Table 2:** Main molecules found in vetiver essential oil from Madagascar, India, the Philippines, Ghana, Guatemala, Haiti, and Indonesia.

Countries	Compounds (%)						
	(E)-Isovalencenol	Khusimol	α-Vetivone	β-Vetivone	Vetiselinenol	β-Vetivenene	Zizanoic Acid
Madagascar	3.3	6.93	5.73	11.46	2.75	4.5	5.16
India	2.4	26.7	2.6	2.8	1.7	12.2	1.0
Indonesia	2.78	10.66	4,25	7.31	0.42	7.13	1.84
Filipinas	4.2	22.08	2.85	7.24	2.15	2.01	1.55
Guatemala	7.1	20.3	2.7	0.6	1.6	2.1	27.2
Haiti	14.62	11.57	6.28	4.45	5.79	2.03	1.60
